# A dual-functional nanogold tablet as a plasmonic and nanozyme sensor for point-of-care applications†

**DOI:** 10.1039/d5na00082c

**Published:** 2025-03-20

**Authors:** Zubi Sadiq, Seyed Hamid Safiabadi Tali, Maryam Mansouri, Sana Jahanshahi-Anbuhi

**Affiliations:** a Department of Chemical and Materials Engineering, Gina Cody School of Engineering and Computer Science, Concordia University Montréal Québec Canada sana.anbuhi@concordia.ca

## Abstract

Point-of-care (POC) devices provide on-site disease diagnosis, particularly in resource-limited settings. Despite considerable progress in POC testing, the availability of commercial devices remains limited, primarily due to challenges in detection sensitivity and portability. Furthermore, advancements in existing POC devices are essential to better meet the needs of end-users. Herein, we present a colorimetric dual-functional tablet sensor using dextran-gold nanoparticles (dAuNPs) to detect and quantify uric acid and glucose levels in urine. Our tablet sensor combines the plasmonic and nanozyme properties of dAuNPs, resulting in highly sensitive detection of both biomarkers. Interestingly, we fabricated the nanogold tablet directly from the dAuNP solution without the addition of any external stabilizer or tablet-forming reagent, thus naming it a direct tablet. An enzyme-free approach was employed for uric acid detection, providing a wide detection range of 0.00187–7.8 mM and a low detection limit of 0.0037 mM, attributed to the hydrogen bonding between dextran and uric acid. On the other hand, the unique nanozyme properties of dAuNPs exhibited exclusive POx-mimetic activity for glucose detection (*K*_m_ = 0.106 mM and *V*_max_ = 369.72 mM min^−1^), with a lower detection limit of 0.625 mM. Our dual-functional tablet offers exceptional substrate selectivity for the colorimetric–chromogenic assay of both uric acid and glucose. This dual-functionality not only provides a highly sensitive, selective, and cost-effective detection strategy for resource-limited settings but also introduces a new avenue for designing customizable plasmonic-nanozyme nanogold tablet sensors as a powerful tool for rapid diagnosis.

## Abstract 2

This work provides new insights into sensor technology by introducing a stable, portable, and user-friendly tablet platform for uric acid and glucose detection, utilizing plasmonic and nanozyme properties of dextran-gold nanoparticles.

## Graphical abstract

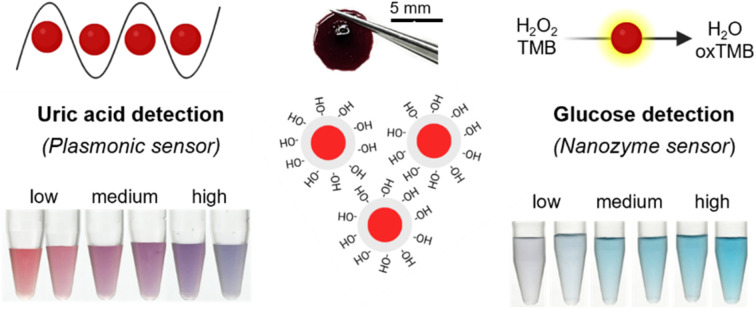

## 1 Introduction

The tablet platform presents a versatile and attractive tool for point-of-use detection across various fields, including the food industry, environmental monitoring, forensic analysis, and disease diagnostics.^1^ Tablets containing solid reagents can be produced through direct compaction of powder mixtures, while liquid reagents are encapsulated by polysaccharides using a drop-casting method. For instance, Udugama *et al.* developed compressed tablets by blending lyophilized powder with mannitol sugar, chosen for its bulking properties, to create chemical diagnostic assays such as nucleic acid and protein tests.^1^ In the drop-casting technique, pioneered in 2014 by our group, different detection reagents, including enzymes and their substrates, and nanoparticles are encapsulated within polysaccharides such as pullulan and dextran to give tablets.^2^ This approach enabled the release of reagents for point-of-use applications such as pesticide and hypochlorite detection in water, as well as glucose and cysteamine detection in bio-fluids.^3–6^

Compressed tablets offer benefits such as high throughput and enhanced dissolution rates, but uniform mixing of reagents necessitates either specialized equipment or labor-intensive processes, along with the use of molds for fabrication. In contrast, the drop-casting approach is simpler, requiring only the pipetting of solutions with pre-optimized concentrations. Overall, tableting technology allows for customizable tablets that hold premeasured quantities of components. Furthermore, labile reagents can be preserved within tablets, preventing reagent degradation due to hydrolysis or oxidation. The tablet form also facilitates safe transportation of chemicals, while making it a user-friendly detection platform for point-of-care (POC) applications.

Hyperuricemia, a condition characterized by elevated uric acid levels, can be measured using both enzyme-based and enzyme-free methods.^7^ While enzyme-based methods are sensitive, they are also indirect and expensive, relying on the uricase enzyme to catalyze uric acid, with the resulting hydrogen peroxide (H_2_O_2_) detected to quantify uric acid concentration.^8,9^ In enzyme-based approaches, electrochemical sensing in sweat and saliva samples is frequently employed, utilizing materials such as uricase embedded zeolitic metal azolate framework-7,^10^ silver nanowire-Prussian blue composite aerogels,^9^ and uricase-immobilized paper.^8^ These methods, however, necessitate labor-intensive preparation of the conducting electrodes and require an electrochemical workstation for signal read-out. On the other hand, enzyme-free methods, though less explored, pose a greater challenge due to their dependence on direct chemical interactions between uric acid and sensing agents.^11,12^ In one such approach, uric acid was detected using 2-thiouracil functionalized gold nanoparticles (TU-AuNPs), where hydrogen bonding and π–π interactions between TU-AuNPs and uric acid served as molecular recognition elements. The plasmonic properties of TU-AuNPs enabled colorimetric detection.^12^ However, the high concentration of 2-thiouracil used during functionalization caused self-aggregation of the particles, reducing their stability and limiting their utility in uric acid sensing. Conversely, a high concentration of uric acid led to an opposite effect due to anti-aggregation or etching behavior resulting in blue to red coloration.^11^ Hence, this sensor requires extensive optimization and careful observation of the detection mechanism.

Glycosuria, characterized by elevated levels of glucose in the urine, occurs when blood glucose concentration surpasses the renal threshold or when the renal tubular reabsorption capacity is diminished. In such cases, excess glucose is excreted into urine without being metabolized by the body. Therefore, monitoring urine glucose levels is crucial for diagnosing diabetes and assessing potential declines in kidney function.^13^ The hybrid glucose oxidase (GOx)/horseradish peroxidase (HRP) assay remains the gold standard for glucose detection. Recently, our group encapsulated both enzymes (GOx and HRP) within a dextran-based tablet sensor for detecting glucose in urine.^14^ In another study, we replaced HRP with an inorganic enzyme-mimic nanomaterial—pullulan-gold nanoparticles (pAuNPs)—which exhibited peroxidase-like nanozyme activity in the oxidation of 3,3′,5,5′-tetramethylbenzidine (TMB) during glucose assay.^3^ This tablet sensor demonstrated potential as a point-of-care (POC) tool for single-analyte detection, though further optimization is needed to advance the technology. In another report, a bifunctional sensing platform composed of polylactic acid and polyethylene glycol fiber mats has been developed for detecting urinary glucose in the range of 1.0 to 6.0 mM.^15^ However, the electrochemical deposition of Prussian blue nanoparticles on the treated mat involves complex pretreatment, complicating the sensing process. Given these limitations, there is an increasing demand for a more user-friendly glucose assay in POC settings. Urine, being a non-invasive sample that maintains its integrity over time, presents an ideal biofluid for POC glucose monitoring, offering both convenience and practicality.

Dual-detection sensors developed for monitoring both uric acid and glucose must exhibit sensitivity within physiologically relevant ranges, with limits of detection (LoD) low enough to differentiate between pathological conditions such as hypouricemia and hypoglycemia and normal levels. In urine, the physiological range for uric acid is 1.40–4.44 mM,^16^ while glucose levels should not exceed 2.8 mM (see Table S1† for details).^17^ Maintaining healthy uric acid and glucose levels is essential to prevent conditions such as kidney stones, gout, diabetes, hypertension, and cardiovascular diseases.^18^ Dual detection of uric acid and glucose has been employed using carboxyl functionalized multiwall carbon nanotubes and Prussian blue-glucose oxidase composite electrodes printed on a rubber glove.^18^ This electrochemical sensor offers simultaneous detection but involves lots of chemicals and requires time-consuming multistep procedures to prepare the electrode. In another study, luminescent sweat tape was introduced to detect uric acid and glucose using enzyme-embedded gold nanoclusters wrapped with MnO_2_ nanosheets as the sensing probe.^19^ Utilization of uricase and GOx besides the requirement of an external light source for signal read-out makes this sensor less practical. Such wearable biosensors are not ideal in POC settings. Therefore, a simple dual-functional POC device capable ofidentifying both hyperuricemia and glycosuria are crucial for tracking an individual's overall health profile.

**Fig. 1:**
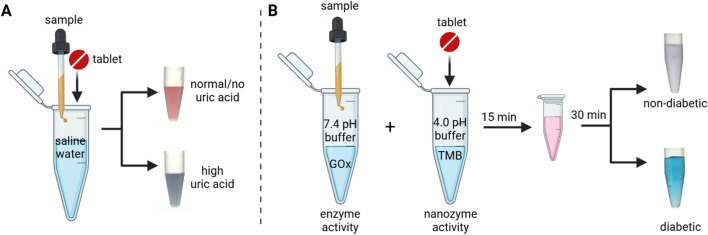
Assay workflow with a dual-functional tablet sensor for uric acid and glucose detection in urine samples. (A) The tablet acts as a plasmonic sensor showing a blue color read-out due to the aggregation of dAuNPs when uric acid is high; (B) the same tablet acts as a peroxidase mimetic nanozyme showing a blue color read-out by oxidizing a chromogenic substance when the glucose level is high.

In our previous work, we demonstrated that dextran-gold nanoparticles (dAuNPs) could be encapsulated in the tablet form to simplify assay procedures and eliminate the need for specific storage conditions.^20^ These customizable tablets were successfully applied for in-field detection of hypochlorite,^4^ as well as point-of-care detection of glucose and cysteamine.^3,21^ In this study, we present the first implementation of a dual-functional tablet for the detection of two analytes: uric acid and glucose. We produced these dual-functional tablets using dAuNPs through a simple, one-pot method without requiring reflux conditions. The dAuNP solution was prepared under ambient conditions and used directly to cast tablets without any additional chemicals. The as-prepared dAuNP solution contained an optimized amount of dextran, sufficient to produce solid, stable tablets capable of detecting both uric acid and glucose colorimetrically. This was achieved by leveraging the plasmonic and nanozyme properties of the dAuNPs (Fig. 1). The colorimetric results were digitized using a UV-vis spectrophotometer, and the concentrations were calculated based on a calibration curve. Our dual-functional tablet proved to be highly efficient for both plasmonic and nanozyme-based sensing, paving the way for future designs of POC diagnostic devices.

## 2 Experimental section

### 2.1 Chemicals and instruments

Full details are provided in the ESI file.†

### 2.2 Synthesis of dAuNPs and formation of direct and indirect tablets

The dAuNPs were prepared according to a previously reported method with considerable modifications.^22^ First, dextran (400 mg) was dissolved in 20 mL of alkaline water (1 M NaOH, 300 μL) under magnetic stirring followed by the addition of HAuCl_4_ (1 mM, 13.8 μL). The resulting mixture remained at room temp (20 °C) under stirring for 10 min to prepare dAuNP colloidal solution. The tablet was fabricated directly from this colloidal solution using the drop-casting method as per our previously established protocols.^20^ The tablet produced from this method is called a direct tablet. We also produced indirect tablets following our pre-established procedure.^4^ In short, the dAuNP solution was synthesized under reflux conditions using HAuCl_4_ (1 mM, 17.22 μL in 25 mL water), dextran solution (5%, 50 μL), and NaOH solution (1 M, 50 μL). Next, an additional amount of dextran (497 mg) was added post-synthetically to the gold solution followed by tablet casting. The tablet produced from this method is called an indirect tablet. One direct or indirect tablet is formed by drop casting 100 μL of dAuNP solution.

### 2.3 Procedure for the detection of uric acid

To optimize the experimental conditions, the impact of different concentrations and volumes of the agglomerating agent (NaCl) was studied. A mixture of 100 μL NaCl solution (0.5, 1.0, 1.5, 2.0, 2.5, and 3.0 M) and 100 μL dAuNP solution was prepared. Also, 30, 50, 100, 150, and 200 μL of aqueous NaCl (2 M) were added to agglomerate the dAuNP solution. After determining the optimal NaCl concentration and volume of NaCl, the uric acid amount was determined using the plasmonic characteristics of the dual-functional tablet. The stock solution of uric acid (125 mM) was prepared by dissolving 126 mg of uric acid in KOH (1 M, 2 mL) followed by the addition of deionized water (4 mL). A serial dilution (up to 1.8 μM) was used in this study. In a micro tube, 100 μL of the standard uric acid solution was mixed with 50 μL of the agglomerating agent solution (2 M NaCl), followed by the addition of 200 μL of deionized water and one direct tablet. The color of the dAuNP solution gradually changed from bright red to purplish blue, indicating nanogold particle agglomeration. A UV-vis spectrometer was then used to detect uric acid in the solution. The excitation wavelengths were 520 and 650 nm. An indirect tablet and dAuNP solution were also used for uric acid determination. The selectivity of the method for uric acid detection was evaluated by adding a series of potential interfering substances (100 mM) including inorganic ions (*i.e.*, sodium, potassium, ammonium, magnesium, calcium, chloride, phosphate, and sulfate), and small molecules (*i.e.*, maltose, fructose, lactose, trehalose, urea, glucose, and ascorbic acid) to the sensing system in the absence of uric acid according to the above-mentioned procedure.

### 2.4 Procedure for the detection of glucose

Initially, the TMB solution (0.41 mM, 300 μL) was added with H_2_O_2_ (0–500 mM, 100 μL) to acetate-phosphate buffer (pH 4.0) along with one direct tablet. The mixture was incubated at 37 °C for 30 min before recording absorbance of the solution at 652 nm. Next, glucose sensing was performed in a chromogenic detection system by incubating the GOx and POx-mimetic nanozyme separately. First, GOx (20 μL) and different glucose concentrations (0–20 mM, 80 μL) were added into 200 mL of phosphate buffer (pH 7.4) while one direct tablet as the POx-mimetic nanozyme and TMB (300 μL) were added into 300 mL of citrate-phosphate buffer (pH 4.0) in a separate microtube. Both microtubes were incubated at 37 °C for 15 min. Next, the contents of both microtubes were mixed and incubated at 37 °C for another 30 min. Finally, 200 μL of the reaction solution was added to a 96-well plate, and the absorption at 652 nm was recorded in a microplate reader. A similar experiment was repeated with an indirect tablet and dAuNP solution. To investigate the selectivity of glucose, different aqueous solutions (100 mM) of inorganic ions (sodium, potassium, ammonium, magnesium, calcium, chloride, phosphate, and sulfate) and small molecules (maltose, fructose, lactose, trehalose, urea, ascorbic acid, and uric acid) were chosen as the interfering substances.

### 2.5 Analysis of real samples

This work was carried out with the consent of Concordia University's Institutional Review Board, with approval number SC5823 and BioPermit number B-SJA-22-01. Additionally, both written and verbal informed consent was obtained from the subjects. The real urine samples from a female and a male volunteer were treated by centrifugation (14 000 rpm, 15 min) before detection. Uric acid with different dosages (low to high concentrations) was added to real samples to carry out the spiking experiment. Similarly, glucose was spiked to the urine samples and the detection procedure was followed as mentioned in Section 2.4. The method was validated by determining the percent recovery (% *R*) and percent relative standard deviation (% RSD) of the results.

## 3 Results and discussion

### 3.1 Synthesis and characterization

**Fig. 2:**
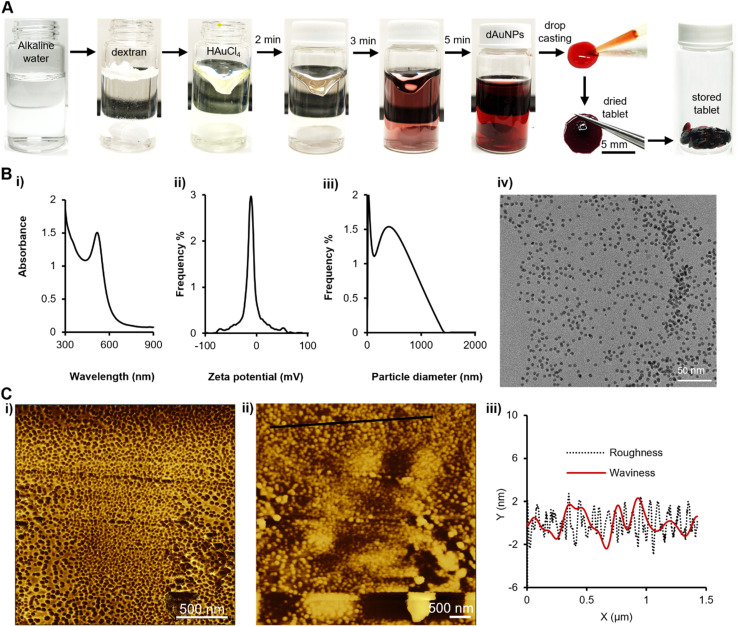
Preparation and characterization of a dual-functional tablet. (A) The dAuNP solution is synthesized at room temperature using a chemical reduction method followed by tablet formation directly from the colloidal solution through the drop casting technique; (B) morphology characterization of nanomaterials in the tablet using (i) the UV-vis spectrum, (ii) zeta potential, (iii) hydrodynamic size of particles, and (iv) TEM analysis; (C) AFM images showing the surface morphology of the direct tablet: (i) the phase trace image showing the dispersed state of nanoparticles, (ii) the height trace 2D topographic image, and (iii) height distribution as surface roughness and waviness in a graph along the black line area in image (ii).

A colloidal dextran-gold nanoparticle (dAuNP) solution was prepared using a simple one-pot, one-phase wet-chemical reduction method and subsequently utilized to fabricate tablets by two methods: direct and indirect. The dAuNP solution for the direct tablets was synthesized at room temperature using hydrogen tetrachloroaurate (HAuCl_4_) and dextran powder in an alkaline aqueous medium (Fig. 2A). Notably, we did not observe particle formation without the addition of sodium hydroxide, which served as the initiator for Au particle formation. The absorption value of the dAuNPs gradually increased with reaction time, with the suspension turning wine red, reaching maximum absorption after 10 minutes, which remained unchanged thereafter.

During the first 2 minutes, the reduction of the gold salt by dextran results in the formation of gold nuclei, which then begin to aggregate. Particle growth occurred through both aggregation and the deposition of additional gold atoms onto the particles.^23^ Over the next 3 minutes, the degree of aggregation increased, resulting in a bright red solution, with stable dAuNPs forming shortly thereafter and remaining unchanged. Dextran played a dual role in the synthesis, acting as both a reducing agent and a stabilizer, preventing agglomeration of the Au particles. As per the IUPAC definition, an agglomeration refers to a situation where dispersed particles are bound by weak physical interactions, resulting in phase separation through the development of precipitates bigger than colloidal size, with the entire process being reversible. On the other hand, aggregation is characterized by the presence of tightly bound colloidal particles, and the clustering process is irreversible.^24^ Aggregation or agglomeration is a dynamic process influenced by various elements, including temperature, light, the surrounding environment, and the chemical properties of the surface ligand. The solution-phase synthetic approach prevents agglomeration through surface functionalization and regulation of surface charge.^25^

The ratio of gold to dextran controlled the size variation of the dAuNPs, with higher dextran concentrations producing smaller particles. Additionally, dextran's tablet-forming properties allowed for the direct formation of tablets from the dAuNP solution, provided that the colloidal solution contained at least 2% dextran (w/v). For our synthesis, we used 2% dextran, maintained a basic pH (∼8), and successfully fabricated solid tablets directly from the dAuNP solution. The concentration of the dAuNP solution was estimated to be ∼261 nM by Beer's-Lambert law using a UV-vis spectrophotometer based on an approximate extinction coefficient (*ε*) of 8.56 × 10^6^ M^−1^ cm^−1^ at 520 nm for 5 nm particles.^26^ The weight of a direct tablet is 1.8 ± 0.2 mg. The resonance absorption band of the dAuNP solution appeared at 520 nm with a narrow size distribution, indicating the monodispersity of the particles. This result was consistent with transmission electron microscopy (TEM) images, which revealed a uniform distribution of spherical Au particles with a size of 5 nm. The zeta potential of the Au species in solution was −11.18 mV, and the hydrodynamic size was measured to be 276 nm (Fig. 2B).

We captured high-resolution surface images of a direct tablet without stains using AFM scanning probe microscopy, as shown in Fig. 2C. The images showed that the nanogold particles were fully dispersed throughout their surroundings and almost uniformly distributed throughout the solid tablet, as evidenced by the phase trace (2Ci). The phase trace image indicates that the particles have a spherical shape, as confirmed by the TEM image. The two-dimensional height profile of a direct tablet demonstrates surface roughness and waviness attributes. The maximum roughness is 2.7 nm and maximum waviness is 2.3 nm. The dextran matrix stabilizes the dAuNPs due to the interaction between the gold nanoparticles and dextran's free alcoholic hydroxyl groups.^20^ This interaction enhances the stability and dispersion of the dAuNPs. So, AFM analysis showed that the particles are fully spread on the polysaccharide surface. As previously reported, the morphology of the Au particles in the solid tablet remained consistent with those in solution, demonstrating the usefulness of this method for sensing and detection applications.^20^ Our approach provides a direct method for tablet formation, eliminating the need for post-synthetic mixing, and making it a straightforward and efficient alternative to solution-phase dAuNPs.

In contrast to direct tablet preparation, reflux conditions were required to synthesize the dAuNP solution for indirect tablets, which were formed after the post-synthetic addition of dextran to the dAuNP solution (Fig. S1†). Initially, 0.01% dextran was employed as both a reducing and capping agent in an alkaline medium to synthesize the colloidal dAuNP solution. Dextran, a reductive polysaccharide with abundant hydroxyl and ether functionalities, surrounds the Au ions, providing stabilization and preventing agglomeration. Dextran chains provide a repulsive surface capping layer around Au nanoparticles. If the capping layer is disrupted, the dAuNPs will agglomerate to form aggregates.^27^ During the reaction, HAuCl_4_ is reduced by dextran, leading to the formation of stable dAuNPs.

Subsequently, additional dextran was added to the synthesized dAuNP solution to achieve an overall concentration of 2%. This post-synthetic addition of dextran is essential for the formation of a rigid solid tablet, referred to as an indirect tablet. The formation of such indirect tablets has been reported in our previous work.^4^ The values of zeta potential and hydrodynamic size in indirect tablets primarily depend on the concentration of dextran in dAuNP solution. With increasing dextran contents in the nanogold colloidal solution, the zeta potential decreases and hydrodynamic size increases. The as-synthesized nanogold colloidal solution with 0.01% dextran has a zeta potential of −41.83 mV and a hydrodynamic diameter of 79.95 nm. The post-synthetic addition of 1.99% dextran shifts the values to −10.80 mV and 292.30 nm. The decrease in negative charge at the surface of dAuNPs results from external neutral dextran that gradually establishes a diffuse layer around the dAuNP surface, partially neutralizing their negative charge. The increased hydrodynamic size results from the thickening of the dextran layer surrounding the Au particles, as previously reported by our group.^4^ Optimal sensitivity in detection is attained with reduced dextran content.^4^ This study utilizes a 2% dextran concentration in colloidal solution, which is the minimum necessary for tablet casting in both direct and indirect methods. The distinction between direct and indirect tablets lies in the incorporation of dextran; direct tablets include the entire amount of dextran all at once, whereas indirect tablets involve a two-step addition, with 0.01% dextran introduced during synthesis and 1.99% added after synthesis. The zeta potential and hydrodynamic diameter values are comparable in both tablets.

It is noteworthy that the position of the maximum absorption peaks remained unchanged, regardless of the preparation method of the dAuNP solution. Furthermore, the sizes of the dAuNPs were difficult to distinguish using UV-vis spectra when both samples were synthesized in aqueous media with dextran as the capping agent. In this work, dextran-coated Au particles require no further surface modification for sensing applications. As a result, dAuNPs act as fully functional nanoprobes and are well-suited for sensor fabrication, offering an efficient platform for various sensing applications. Throughout this study, we used tablets created using the previously mentioned direct method, unless otherwise mentioned. Both the direct and indirect tablets exhibit high inertness to atmospheric conditions and can be stored at room temperature, maintaining stability for over a year, as previously reported.^3,20^ The distinguishing features of direct and indirect tablets can be found in Table S2.†

### 3.2 Mechanistic insights into the dual-functional tablet sensor

**Fig. 3:**
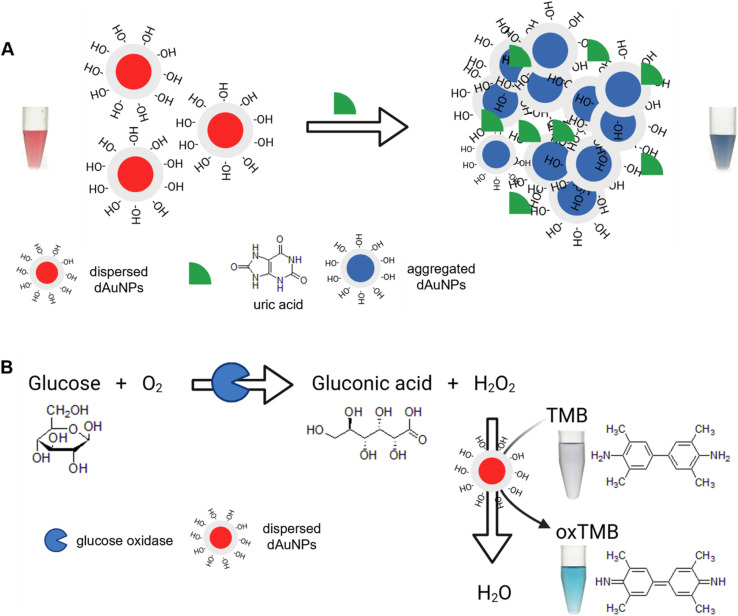
Schematic mechanism for the dual-functional tablet sensor. (A) The tablet acts as a plasmonic sensor detecting uric acid; (B) the tablet acts as a nanozyme sensor detecting glucose.

Gold nanoparticles are an excellent nanoscale material for colorimetric detection due to their unique optical properties.^28–30^ After successful fabrication and thorough characterization of the tablets, the dAuNPs have been extensively studied for their sensing and catalytic capabilities. Our dual-functional tablet incorporates ∼5 nm spherical dAuNPs, which exhibit both plasmonic response and peroxidase (POx)-mimetic activity for the detection of uric acid and glucose, respectively. We leveraged these dual-functionalities in our sensing system. The plasmonic colorimetric response is based on a uric acid-induced color change in the dAuNPs, as illustrated in Fig. 3A. The carbonyl moieties of uric acid form hydrogen bonds with the hydroxyl groups of dextran, causing dAuNP aggregation. The existence of π–π interactions and hydrophobic forces further reduces the interparticle distance, promoting this aggregation.^12^ Additionally, the ionic nature of salt (*i.e.*, sodium chloride) enhances this aggregation. The aggregation of nanoparticles alters their optical properties, causing a visible red-to-blue color shift that aids in the colorimetric identification of target agents. The gold nanoparticles undergo agglomeration at lower analyte concentrations but aggregation at higher concentrations, as reported in the literature.^31^ The visible color change in the solution corresponds to the change in the spectral profile of dAuNPs, which allows for the quantification of uric acid. This method is highly sensitive and selective, effectively distinguishing uric acid even in the presence of coexisting interferents in urine samples.

For glucose detection, the dual-functional tablet operates as a nanozyme sensor *via* a chromogenic reaction with TMB (Fig. 3B). The peroxidase-like activity of dAuNPs catalyzes the oxidation of the TMB substrate in the presence of hydrogen peroxide (H_2_O_2_). H_2_O_2_ and TMB adsorb on the surface of dAuNPs and their proximity on the surface enhances the reaction efficiency. Also, nanogold particles' intrinsic nanozyme activity depends on the nature of their surface charges, which can be either positive or negative. The negative surface charge significantly enhances the affinity of nanoparticles for the peroxidase substrate (TMB) *via* electrostatic attraction under mildly acidic conditions.^3^ Initially, H_2_O_2_ decomposes into reactive oxygen species (*e.g.*, hydroxyl radicals (·OH) and superoxide anions (O_2_·^−^)), which oxidize TMB due to the two readily oxidizable amino groups on its benzidine core. The negative charge on dAuNPs, owing to the presence of the hydroxyl group, attracts the positively charged amino group of oxTMB, thereby facilitating the nanozyme process. The resulting oxTMB is a colored product, formed through a one-electron route which shows an absorption peak at 652 nm.^3^ The intensity of this peak directly correlates with the amount of H_2_O_2_, which in turn reflects the glucose concentration in the sample. Our dual-functional tablet sensor demonstrates excellent performance in detecting both uric acid and glucose. The plasmonic response is driven by the high molar extinction coefficient of dAuNPs, while the nanozyme activity is enhanced by their small particle size, ensuring a highly efficient and sensitive detection method for both analytes.

### 3.3 Dual-functional tablet sensor for uric acid detection

This section focuses on the optimization studies and the detection of uric acid using the plasmonic properties of a dual-functional tablet, followed by an analysis of selectivity and spiking results.

#### 3.3.1 Optimization studies

**Fig. 4:**
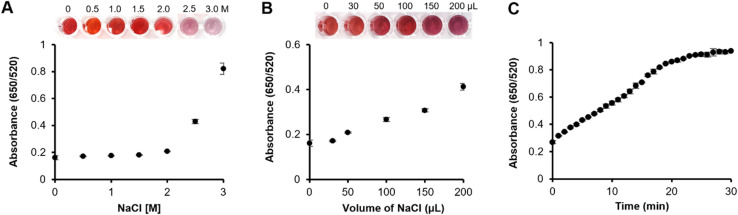
Optimization of an assay for uric acid detection using a direct tablet. (A) Varying concentrations of NaCl were tested; (B) different volumes of NaCl were used; (C) kinetic study.

The direct tablet was employed to optimize reaction conditions, including variables such as the concentration and volume of NaCl, as well as the kinetics of the uric acid assay (Fig. 4). Optimal experimental conditions were determined by measuring absorbance at 520 nm and 650 nm, which represent the dispersed and aggregated states of Au nanoparticles, respectively. It was found that 2 M NaCl (100 μL) did not cause a visible color change in the dAuNP tablet, indicating that this condition did not interfere with the uric acid assay but rather enhanced signal intensity. A salt concentration >2 M induced aggregation without an analyte, and was hence unsuitable for the detection system. Next, a volume of 2 M NaCl was optimized to obtain the best working ratio. A volume of 100 μL of NaCl was selected to enhance the signal intensity. Finally, the reaction kinetics revealed a 20 minute window for maximum color development (Fig. 4).

It is important to mention that we have examined the impact of varying pH levels on uric acid detection. We have evaluated various buffer solutions with pH levels from neutral to alkaline, including phosphate buffer at pH 7.0, Tris–HCl buffer at pH 7.4, and KH_2_PO_4_–NaOH buffer at pH 8.2. Since acidic conditions cause aggregation of the dAuNP tablet, we refrained from utilising an acidic buffer. None of the buffer systems were effective for uric acid detection, as we did not see a gradual colorimetric response with varying concentrations of uric acid. The failure of the buffer system in this assay is attributed to the significant pH variation induced by varying amounts of uric acid in the test solution. The pH of a standard uric acid solution is ∼12, while the pH of the dAuNP colloidal solution is ∼8 due to its alkaline-assisted synthesis. The pH of the test solution remained ∼11 up to a uric acid content of 1.95 mM. However, at higher uric acid concentration, the pH increased, reaching ∼13 at 62.50 mM. This significant pH change within the uric acid concentration range makes uric acid detection impractical in a buffered system.

#### 3.3.2 Quantitative analysis of uric acid

**Fig. 5:**
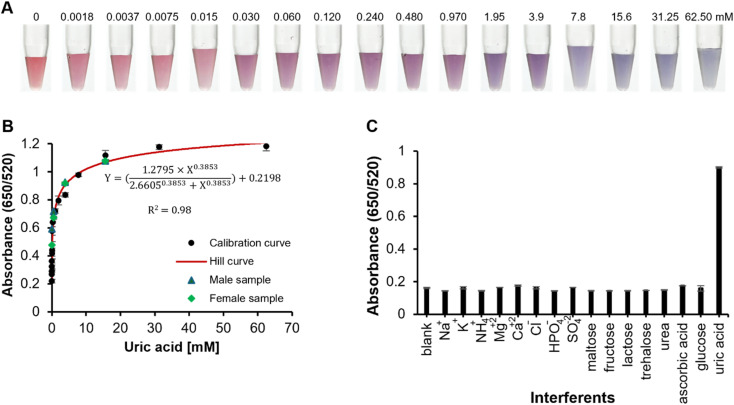
Detection of uric acid using a dual-functional tablet sensor. (A) Optimal images of color transition in a reaction vial; (B) a calibration plot indicating a concentration-dependent response of a direct tablet for uric acid along with real-sample spiking results; (C) selectivity of the proposed dual-functional tablet sensor.

A calibration plot for uric acid detection was generated using the absorbance ratio (650/520), as shown in Fig. 5. The bright red color of Au nanoparticles shifts to violet-blue in the presence of uric acid and the optimized amount of NaCl. The dextran chains surrounding the dAuNPs not only provide particle stability but also serve as functional units, facilitating interactions with uric acid. In particular, the carbonyl group of uric acid may form hydrogen bonds with the hydroxyl group of dextran, reducing the interparticle distance and inducing aggregation of the dAuNPs. As shown in Fig. 5B, the absorbance ratio (650/520) increases proportionally with the concentration of uric acid. Sodium chloride enhances the intensity of particle aggregation, improving the sensitivity of the assay. A Hill function was used to fit the data of the calibration curve, giving an *R*^2^ value of 0.98, indicating a strong fit with the data. This confirms that the data indicates a typical saturation behavior, consistent with the sensor's expected performance. The limit of detection (LoD) was calculated as three times the standard deviation of the blank signal added to the mean signal of the blank. The direct tablet sensor demonstrated a working range of 0.00375 to 7.8 mM, with an LoD of 0.0037 mM, which is in the biologically relevant range and makes it a reliable tool for uric acid detection. The ultra-wide working range and low LoD are attributed to the strong plasmonic response of dAuNPs, which amplifies the sensitivity of uric acid detection, resulting in a lower LoD. The use of NaCl as a signal amplifier further improves signal transduction, rendering absorbance changes highly responsive to variations in uric acid concentration. Furthermore, the surface of dAuNPs is optimized with dextran chains to ensure selective binding with uric acid, hence minimizing non-specific interactions and enhancing both the sensitivity and range. The sensor's distinct color change from red to blue, caused by dAuNP aggregation in the presence of uric acid, is easily detectable even at minimal concentrations.

Our proposed uric acid assay is suitable for POC applications. We employed smartphone-assisted quantification of uric acid with ImageJ software to illustrate this. The color intensity of each uric acid working solution was quantified, and a calibration curve was generated, as illustrated in Fig. S2.† A sigmoidal curve was obtained for the concentration range of 0–62.50 mM, with an LoD of 0.0037 mM. A gradual change in color intensity is evident in the logarithmic scale graph (Fig. S2B†). To guarantee precise colour selection and uphold uniformity in the height and angle throughout image acquisition, we utilized our laboratory-constructed imaging apparatus with regulated lighting and a stationary sample holder. Therefore, a dual-functional tablet sensor is applicable for uric acid detection in POC scenarios.

The working range of uric acid detection can be adjusted by utilizing different nanoprobes, such as indirect tablets and as-prepared dAuNP solution. The indirect tablet exhibited a working range of 0.0075 to 15.6 mM, with a limit of detection (LoD) of 0.0075 mM, while the dAuNP solution demonstrated a range of 0.03 to 1.95 mM with an LoD of 0.03 mM (Fig. S3†). Calibration plots include data represented as the mean and standard deviation from three independent measurements.

#### 3.3.3 Selectivity analysis

To assess the selectivity of the dual-functional sensor, several potential interfering substances commonly found in human urine, including various inorganic ions (Na^+^, K^+^, NH_4_^+^, Mg^2+^, Ca^2+^, Cl^−^, HPO_4_^−^, and SO_4_^2−^) and small molecules (maltose, fructose, lactose, trehalose, urea, glucose, and ascorbic acid), were tested at 100 mM concentrations in a detection system without uric acid. As shown in Fig. 5C, none of these interferants affected the absorbance ratio, confirming the high selectivity of the sensor for uric acid.

#### 3.3.4 Validation with real samples

The plasmonic properties of the dual-functional tablet sensor were further utilized for uric acid detection in real urine samples. First-morning urine samples were collected from a male and female volunteer, centrifuged at 14 000 rpm for 15 min, and the supernatant after spiking with 0.48, 3.9, and 15.6 mM uric acid was used for analysis (Fig. 5B). The recovery percentages (% *R*) for male urine samples were 116 ± 2.2, 108 ± 3.3 and 103 ± 1.9, while those for female urine samples were 103 ± 3.1, 111 ± 1.4, and 110 ± 1.7, confirming the assay's accuracy. The assay showed excellent precision and reproducibility, with percent relative standard deviation (% RSD) values below 3% for all samples, each analyzed in triplicate. The spiking analysis confirmed the successful application of the proposed sensor for uric acid detection in real urine samples, showing its potential for clinical applications and daily monitoring. Moreover, this sensor is not limited to detecting uric acid in urine; with the appropriate dAuNP nanoprobe, it can be adapted to detect uric acid in other biofluids. For instance, uric acid detection in serum can be achieved using direct or indirect tablets, as their operational ranges (0.00375–7.8 mM or 0.0075–15.6 mM) align with the required uric acid level in serum (0.120–0.400 mM). This optimal detection capability makes the tablet sensor highly suitable for serum analysis, thereby expanding its utility in POC settings.

### 3.4 Dual-functional tablet sensor for glucose detection

Glucose levels in body fluids serve as crucial biomarkers for various diseases, including diabetes and vascular conditions. Thus, developing nanostructures capable of glucose monitoring is an important strategy for early and accurate disease detection. In this study, we employed the peroxidase (POx)-mimetic properties of a dual-functional tablet to quantify glucose levels.

#### 3.4.1 Optimization studies

**Fig. 6:**
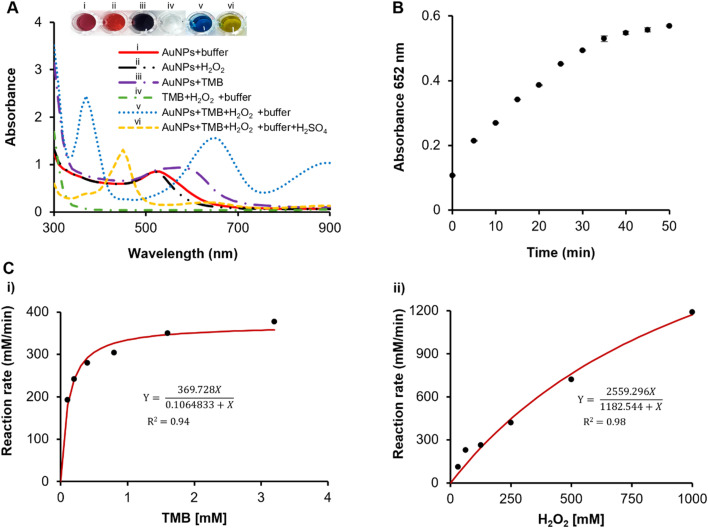
Optimization and kinetic studies for glucose detection using a direct tablet. (A) Absorbance spectra; (B) a graph showing a gradual increase in the concentration of oxTMB with time; (C) steady-state kinetics of a dAuNP tablet.

The optimization of reaction conditions, including varying TMB concentrations, pH, reaction time, and temperature, was performed. TMB concentrations of 0.4, 0.6, 0.8, 1.0, and 1.2 mM were tested, and 0.8 mM was found to be the best amount required for this reaction. Oxidised TMB (oxTMB) exhibited a blue hue at 652 nm, while unreacted TMB remained colourless, which was measured using a UV-vis spectrophotometer. Similarly, both reaction time and temperature were monitored, revealing that an increase in temperature correlates with a decrease in reaction time, as supported by the literature.^3^ A 30 minute incubation at 37 °C produced the optimal results. Glucose oxidase is active within a pH range of 4–8; therefore, the experiment was conducted at pH levels from 3.0 to 8.0 using acetate–phosphate and phosphate buffers. The optimal pH for optimal peroxidase activity in the acetate–phosphate buffer was 4.0, while the optimal pH for glucose oxidase activity in the phosphate buffer was 7.4. The absorbance spectra of dAuNPs in the presence of various reagents are illustrated in Fig. 6A. Fully dispersed dAuNPs exhibit a characteristic peak at 520 nm, which broadens and shifts to 530 nm in acetate buffer (pH 4.0). No change in particle dispersion was observed with 1 M H_2_O_2_. However, TMB induced aggregation of the dAuNPs, marked by an absorbance peak at 570 nm. The POx-mimetic activity of the dAuNPs was confirmed by the appearance of a blue color at 652 nm (*λ*_652_) when TMB was oxidized by H_2_O_2_ (Fig. 6A-v). This catalytic reaction was terminated using a stop solution (*e.g.*, H_2_SO_4_), which produced a yellow color with an absorbance peak at 450 nm (*λ*_450_).

After confirming the POx-mimetic behavior of the dAuNP tablet, we proceeded to calculate the steady-state kinetics of the catalytic reaction using the Michaelis–Mention plots. To provide an in-depth understanding of the POx-mimetic activity of the tablet, the kinetic parameters were assessed by altering the concentrations of TMB and H_2_O_2_. The kinetic parameters, including the Michaelis–Menten constant (*K*_m_) and maximum reaction velocity (*V*_max_), were determined to evaluate the reaction's efficiency. The reaction rates for TMB oxidation were calculated and used to produce standard Michaelis–Menten curves (Fig. 6C). Through nonlinear fitting of the Michaelis–Menten plot, we ascertained the kinetic parameters (*V*_max_ and *K*_m_) using a website (https://www.mycurvefit.com). A high *K*_m_ signifies weak enzyme-substrate affinity, while a low *K*_m_ indicates strong affinity. The calculated *K*_m_ value for the direct tablet is 0.106 mM with TMB, which is lower than that of the pullulan-gold nanoparticle tablet (*K*_m_ = 0.142 mM) (ref. 3) and the natural HRP enzyme (*K*_m_, _TMB_ = 0.434 mM),^32^ indicating a strong affinity of the direct tablet for TMB. However, the *K*_m_ value with H_2_O_2_ was higher (1182 mM) compared to that of HRP (3.7 mM), suggesting that a higher H_2_O_2_ concentration is needed to achieve the maximum activity of dAuNPs, as supported by the literature.^33^ The calculated *V*_max_ with TMB for the direct tablet is 369.72 mM min^−1^. The Michaelis–Menten plots for the dAuNPs displayed a hyperbolic curve when TMB and H_2_O_2_ were used as substrates. These results highlight the rapid catalytic kinetics of dAuNPs, underscoring their potential for POx-mimetic activity in glucose detection.

#### 3.4.2 Quantitative analysis of glucose

**Fig. 7:**
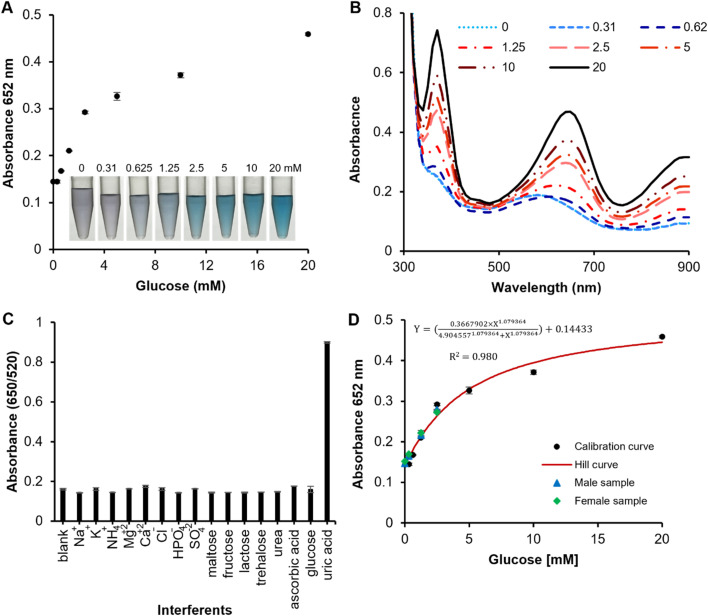
Peroxidase (POx) mimetic nanozyme performances of a dual functional tablet sensor for glucose detection. (A) A calibration curve indicating a concentration-dependent response of a tablet sensor using a TMB substrate; (B) the absorbance spectra indicating a gradual increase in peak intensity with increasing glucose concentration; (C) selectivity analysis; (D) spiking analysis.

Following this, quantitative colorimetric detection of the H_2_O_2_ level is evaluated by using the POx-mimetic activity of a direct tablet. The characteristic absorption peak at 652 nm increases gradually with increasing concentrations of H_2_O_2_ in the presence of TMB as a substrate (Fig. S4†). Subsequently, glucose detection was performed in a two-step reaction process. In the first step, glucose oxidase (GOx) catalyzes the oxidation of glucose into gluconic acid, releasing H_2_O_2_ as a by-product. In the second step, the peroxidase-mimetic (POD-mimetic) activity of the dAuNPs oxidizes TMB in the presence of the generated H_2_O_2_, leading to a detectable colorimetric change. A positive correlation between absorbance intensity and glucose concentration was observed, confirming the method's high sensitivity for glucose detection (Fig. 7). The LoD for the direct tablet was determined to be 0.625 mM with a working range of 0.625–10 mM. A similar working range (0.625–20 mM) was reported by Sun *et al.* for glucose assay using the peroxidase-mimetic activity of core–shell Cu/Au nanoparticles.^34^ Another study recorded a linear range of 0.05–20 mM for glucose assay using Au–Ag heterogeneous nanorods.^35^

On the other hand, smartphone-assisted quantification of glucose was achieved *via* ImageJ analysis (Fig. S5†). Digital photos were captured through a smartphone in a controlled environment at a fixed distance, adhering to the principles of colorimetric research. These results are consistent with those obtained with a UV-vis system (working range of 0.625–10 mM and LoD of 0.625 mM), demonstrating the compatibility of ImageJ analysis for glucose detection. Previously, our group reported glucose oxidase encapsulation in a solid tablet which solves the storage and stability challenges related to enzymes.^14^ The enzyme encapsulated tablets exhibit heat stability up to 60 °C and have been effectively utilized for glucose detection in urine. Thus, tablet-based sensors provide a user-friendly tool for POC applications.^27^ The comparison of the current method with previously reported assays is outlined in Table S3.†

Further evaluations of glucose detection using the indirect tablet and as-prepared dAuNP solution were conducted, with the results presented in Fig. S6.† Both systems demonstrated a proportional linear relationship between absorbance intensity and glucose concentration. The indirect tablet exhibited a working range of 0.31–5 mM, while the as-prepared dAuNP solution had a range of 0.31–10 mM. The working range of the dual-functional tablet is broader compared to the working range (0–6 mM) of our previously reported tablet assay.^14^ These findings highlight the versatility and effectiveness of the dual-functional tablet system for glucose detection across different nanoprobe formats.

#### 3.4.3 Selectivity analysis

Human urine is a complex matrix containing a variety of inorganic salts and organic compounds, which can complicate the precise identification of the target biomarker. Therefore, eradicating any potential interfering substances ensures a reliable signal output. Common strategies to mitigate interference in complex samples include dilution and the use of masking agents. In this study, we centrifuged the urine sample to minimise the matrix effect. The absorbance intensity of oxTMB followed a similar trend to the control when various potential interfering substances (maltose, fructose, lactose, trehalose, urea, ascorbic acid, and uric acid) and inorganic ions (sodium, potassium, ammonium, magnesium, calcium, chloride, phosphate, and sulfate) were tested. This confirms the excellent selectivity of the direct tablet sensor for glucose detection (Fig. 7C).

#### 3.4.4 Validation with real samples

Finally, to evaluate the practical applicability of our proposed glucose assay in real-world scenarios, human urine samples from both male and female volunteers were spiked with glucose concentrations of 0.31, 1.25, and 2.50 mM glucose and tested using the dual-functional tablet sensor. As shown in Fig. 7D, the blue triangles representing male samples indicate that the measured intensities for the blank and spiked samples followed the calibration curve trend, with calculated recovery values of 103 ± 3.1, 105 ± 1.7, and 116 ± 1.3% for the 0.31, 1.25, and 2.50 mM spiked samples, respectively. Similarly, the green diamonds for female samples show the calculated recovery values of 101 ± 3.1, 99 ± 0.9, and 105 ± 2.3%. The assay also demonstrated excellent precision, with % RSD values below 2% for all data points across three replicates. These findings highlight the assay's applicability in complex matrices, underscoring the high feasibility and reliability of the dual-functional tablet sensor for glucose detection in real biological samples, with significant potential for clinical and routine monitoring applications. To develop a complete device suitable for POC settings, an opaque box can be designed and combined with smartphone imaging to offer an affordable, portable, and user-friendly solution, as highlighted in the literature.^36^

## 4 Conclusion

In this work, we highlight the use of dextran-gold nanoparticles (dAuNPs) as a complete nanoprobe, emphasizing their tablet-forming properties and dual-functional colorimetric detection capabilities. The dAuNPs exhibit both plasmonic and peroxidase (POx)-mimetic nanozyme characteristics, which were utilized to develop a dual-functional sensor for the detection of uric acid and glucose in urine, respectively. A concentration-dependent plasmonic response toward uric acid was achieved within the range of 0.00375–7.8 mM, which falls within the biologically relevant range of 1.4–4.44 mM. Similarly, the tablet sensor demonstrated nanozymatic activity in the range of 0.625–10 mM for glucose, suitable for glucose monitoring in urine. Our dual-functional tablet sensor is highly sensitive and selective, and remains stable for over a year. The advantage of this technology lies in its ability to combine both plasmonic and nanozyme properties within a single tablet, enabling portable testing for field use. This tablet-based approach offers a simple and effective method for developing self-monitoring testing kits with visual readouts. The promising performance of this tablet sensor indicates its potential for fast, safe, and convenient monitoring of uric acid and glucose in clinical settings and for self-care testing.

## Data availability

The authors declare that the data supporting our study “A dual-functional nanogold tablet as a plasmonic and nanozyme sensor for point-of-care applications” are available within the article and its ESI.†

## Author contributions

ZS: conceptualization, investigation, methodology, formal analysis, validation, prepared figures, writing – original draft, review and editing; SHST: review and editing, visualization; MM: formal analysis, methodology; SA: conceptualization, project administration, supervision, funding acquisition, resources. All authors read and commented on the full manuscript.

## Conflicts of interest

The authors declare no competing financial interest.

## Supplementary Material

NA-007-D5NA00082C-s001
