# Supplementary material for: A dual-functional nanogold tablet as a plasmonic and nanozyme sensor for point-of-care applications
Source: Nanoscale Adv. 2025 Mar 20;7(10):2967–78. doi: 10.1039/d5na00082c (PMC11960780; doi:10.1039/d5na00082c)
Supplement: NA-007-D5NA00082C-s001 [file NA-007-D5NA00082C-s001.pdf]

Supplementary Information (SI) for Nanoscale Advances.
This journal is © The Royal Society of Chemistry 2025

SUPPORTING INFORMATION

Dual-functional nanogold tablet as a plasmonic and nanozyme sensor for point-of-care applications

Zubi Sadiq[1], Seyed Hamid Safiabadi Tali[1], Maryam Mansouri[1], Sana Jahanshahi-Anbuhi[1*]

[1]Department of Chemical and Materials Engineering, Gina Cody School of Engineering and Computer Science, Concordia University, Montréal, Québec, Canada

[*]Address correspondence to Sana Jahanshahi-Anbuhi, sana.anbuhi@concordia.ca

Table of contents:

- additional experimental details
- additional results including figures S1-S6
- tables S1-S3

ADDITIONAL EXPERIMENTAL DETAILS

Chemicals and instruments

All the chemicals were of analytical grade and used as received. Tetrachloroauric acid (30 wt.% in dil. HCl), trisodium citrate, sodium hydroxide, dextran (100 kDa), uric acid, sodium chloride, urea, thiourea, ascorbic acid, hydrogen peroxide (30 wt.%), glucose, glucose oxidase (GOx) from *Aspergillus niger,* 3,3´,5,5´- tetramethylbenzidine, maltose, lactose, fructose and sulfuric acid were purchased from Sigma-Aldrich. Dimethyl sulfoxide was obtained from Fisher Scientific, Toronto, ON, Canada. Phosphate buffer (100 mM, 7.4 pH) was prepared using monosodium phosphate monohydrate (20.26 mM) and disodium phosphate heptahydrate (79.74 mM). Citrate-phosphate buffer (50 mM, 4.0 pH) was prepared using citric acid monohydrate (100 mM) and disodium phosphate heptahydrate (200 mM). The pH adjustment was performed using HCl and NaOH solutions. The TMB stock solution was prepared by dissolving 1 mg/mL in DMSO followed by preparing a working solution by diluting 1 mL of the DMSO stock with 9 mL phosphate citrate buffer of pH 4.0. The GOx solution (180 U/mL) was prepared in tris buffer (100 mM, 7.4 pH). Artificial urine was purchased from Biochemazone, Leduc, AB, Canada. Real urine samples were obtained from healthy volunteers and analyzed in this study according to the agreement of Concordia University's Institutional Review Board with the approval number SC5823 and the BioPermit number B-SJA-22–01. The UV−vis spectrophotometer (BioTek, Cytation 5, imaging

reader), particle size analyzer (model Litesizer 500, Anton-Paar, Austria), and transmission electron microscopy (Talos L120C, 20−120 kV) are used for characterization of dAuNPs solution. For AFM image analysis (Anton Paar Tosca 400, Austria), a solid direct tablet was fixed to the sample stage onto with the tapping mode in air. An aluminum reflex coated cantilever (thickness: 30 nm, resonance frequency: 285 kHz, curvature height 10-15 μm; radius: <10 nm) was used, and the 600 × 600 pixel images were collected at a line rate of 0.3 lines/s. Image analysis was done using Gwyddion (free, open-source software, version 2.67).

ADDITIONAL RESULTS

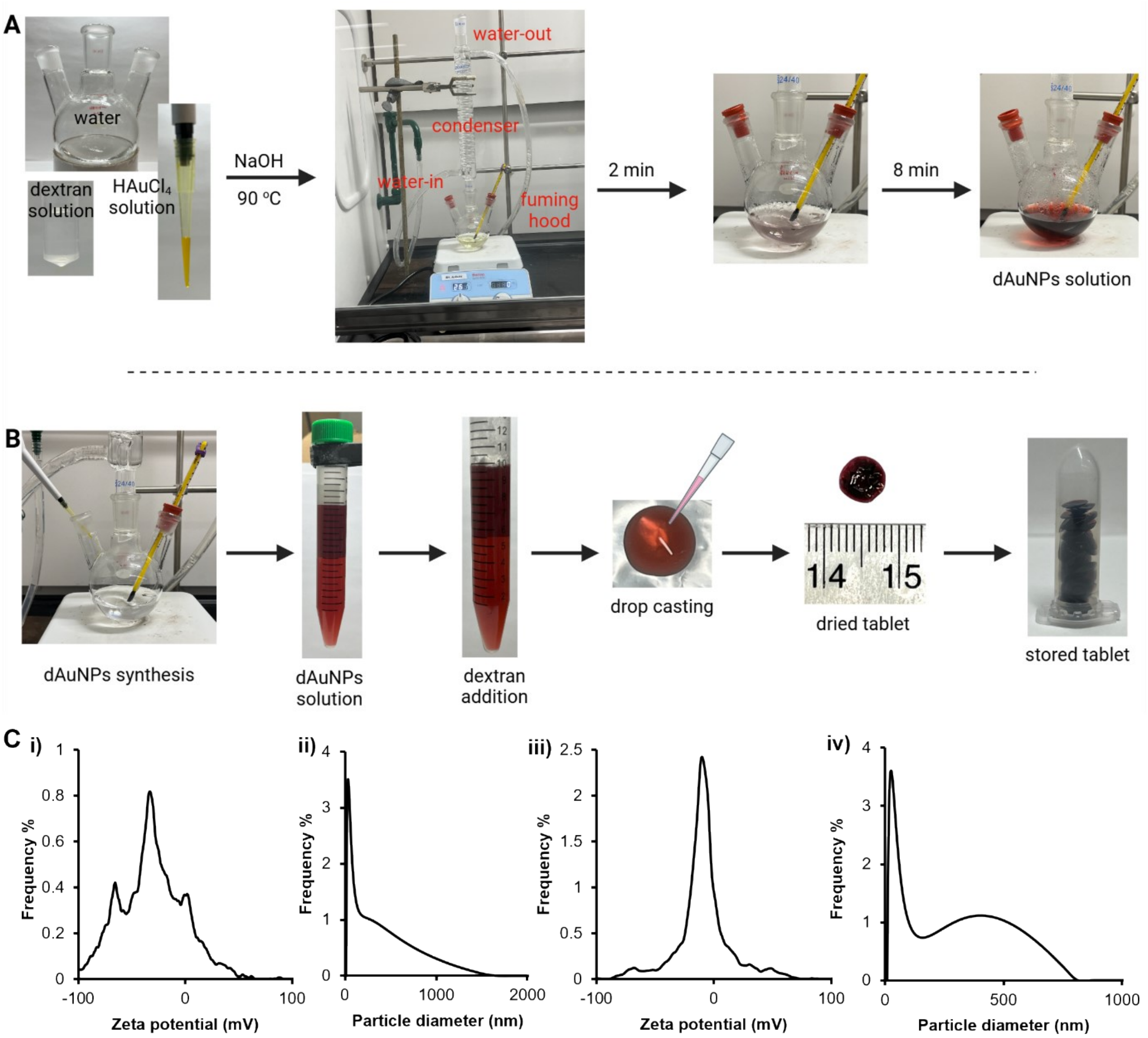

Fig. S1 Synthesis of dAuNPs solution and tablet formation. A) The chemical reduction method is used to produce colloidal dAuNPs; B) Indirect tablets are produced from dAuNPs solution after post-synthetic dextran addition; C) i) Zeta potential, and ii) hydrodynamic size of dAuNPs colloidal solution, iii) zeta potential, and iv) hydrodynamic size of the indirect tablet.

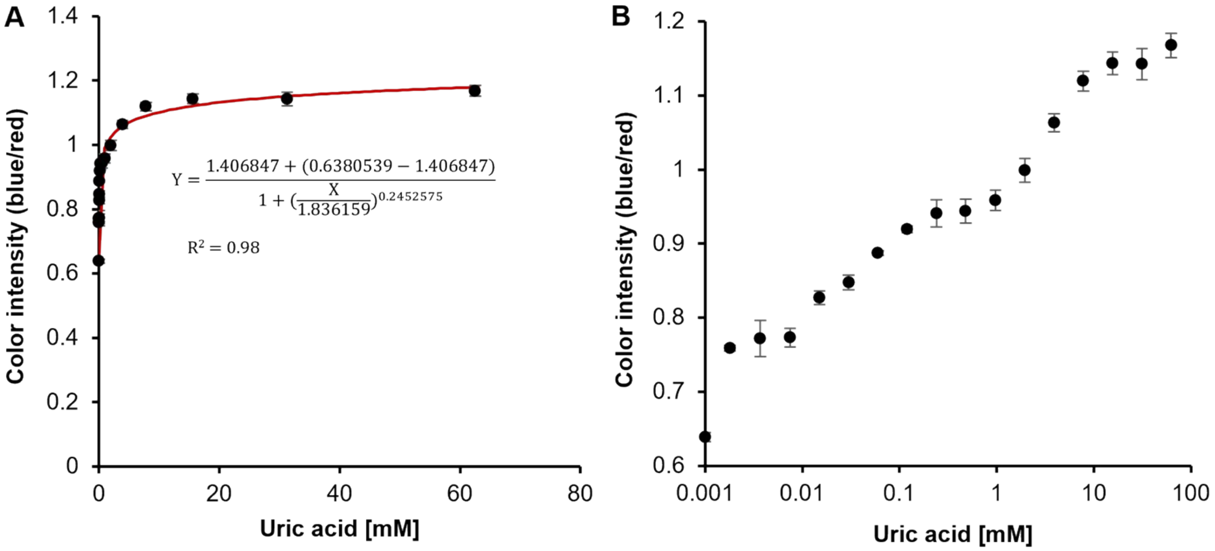

Fig. S2 *ImageJ*-based quantification of uric acid with the direct tablet. A) A calibration graph showing a sigmoidal curve; B) A calibration plot in log scale showing a gradual change in color intensity.

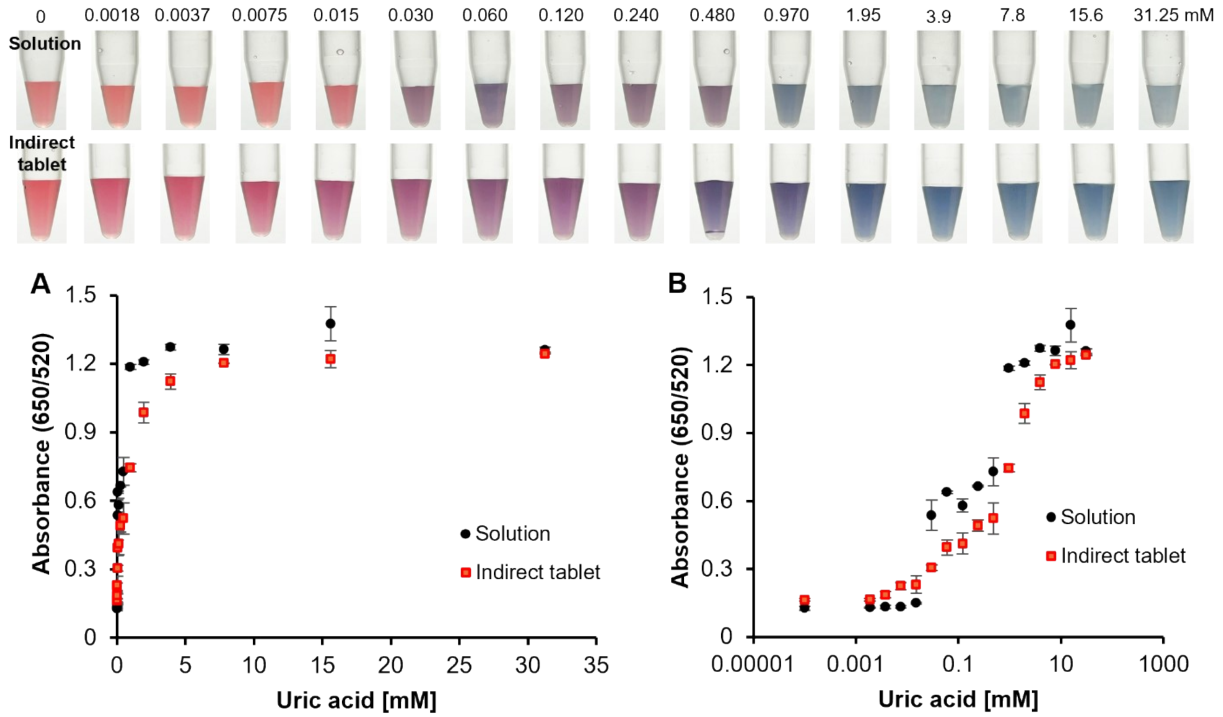

Fig. S3 Calibration curve for the detection of uric acid using indirect tablet and solution. A) The response curve showing a broader working range (0.0075 - 15.6 mM) with an indirect tablet as compared to the solution (0.03 - 1.95 mM); B) A calibration plot in log scale.

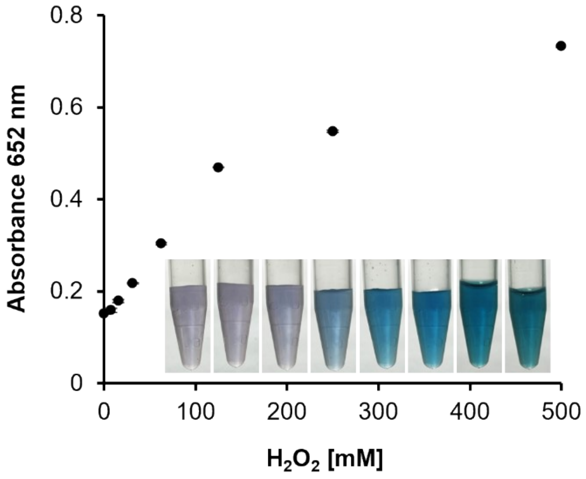

Fig. S4 Calibration curve for the detection of H_2O_2 using a direct tablet.

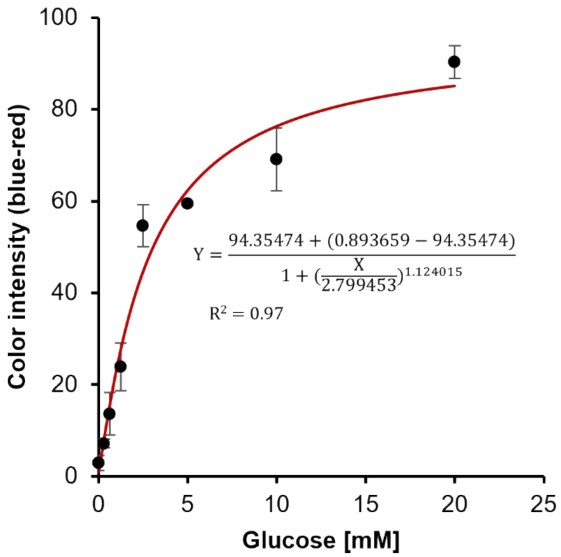

Fig. S5 *ImageJ*-based quantification of glucose with the direct tablet.

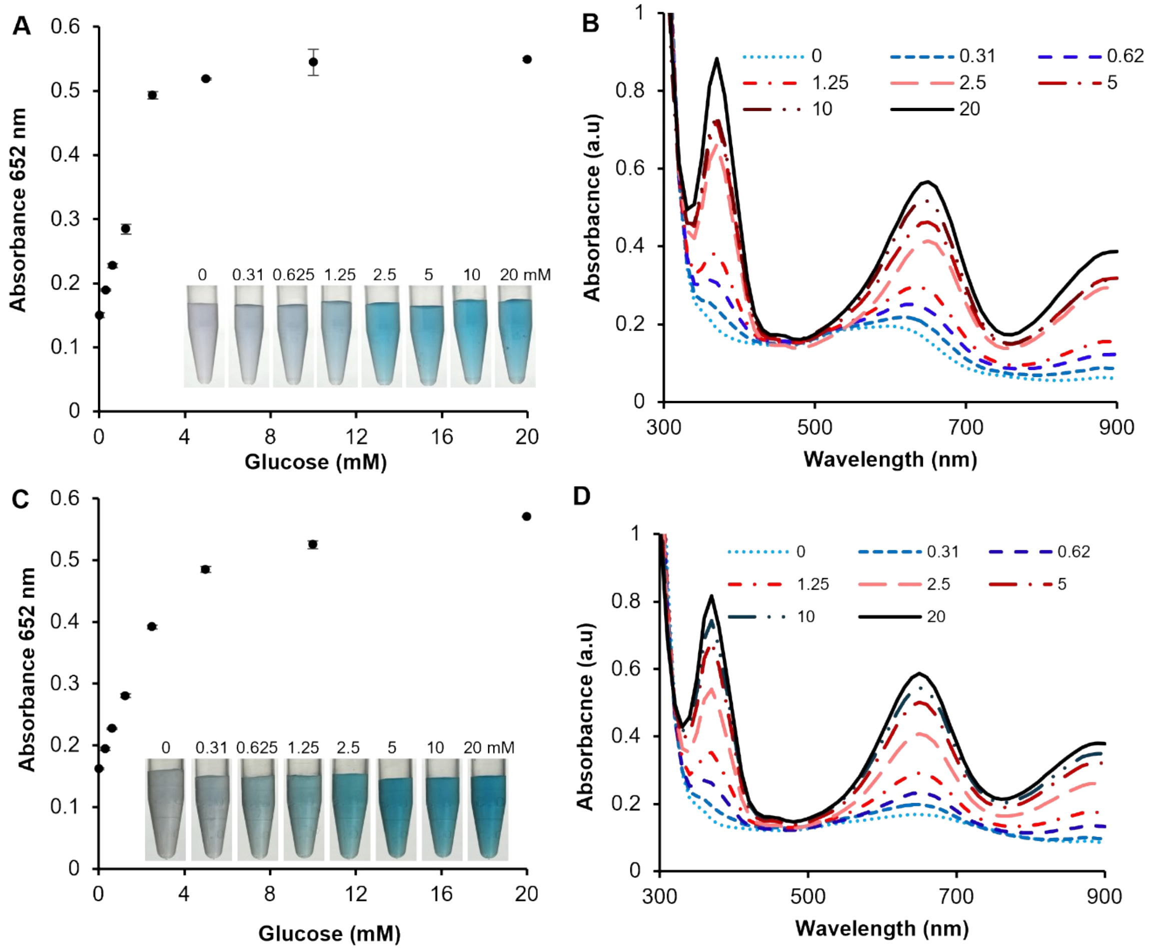

Fig. S6 Calibration curve for the detection of glucose using indirect tablet and solution.

Table S1. Uric acid and glucose levels in different biological fluids

Analytes	Biological fluid	Normal/healthy range (mM)	Ref.
Uric acid	urine	1.4 – 4.44	[1]
	blood (serum)	0.120 – 0.400	[2]
	saliva	0.172 – 0.226	[2]
	sweat	0.020 – 0.025	[3]
	tear	0.025 – 0.150	[4]
Glucose	urine	0 - 2.8	[5]
	blood (serum)	4.0 – 8.0	[6]
	saliva	0.039 – 0.122	[7]
	sweat	0.28 – 1.11	[6]
	tear	0.1 – 0.6	[8]

Table S2. Distinguished features of direct and indirect tablets

**Aspect**	**Features**	**Direct Tablet**	**Indirect Tablet**
Synthesis/ Preparation	Heat	room temperature (20 °C)	reflux temperature (100 °C)
	Time	10 min	60 min
	$HAuCl_4$ (for 25 mL colloidal solution)	1 mM	1 mM
	Dextran concentration	2% (all at once during synthesis)	2% (0.01% during synthesis + 1.99% after synthesis)
	NaOH	1 M, 375 µL	1 M, 50 µL
	Procedure	one-step: directly from solution to tablet	two-steps: an extra step of post-synthetic dextran addition involves
	Stability	>1 year (till to-date)	>4 years (till to-date)
	Storage	room temperature	room temperature
Detection	Particle size	5 nm	13 nm
	Zeta potential	-11.18 mV	-10.80 mV
	Hydrodynamic size	276 nm	292 nm
	Function	suitable for plasmonic and nanozyme sensor fabrication	suitable for plasmonic sensor fabrication
	Dual-functionality	excellent	good

Table S3. Comparison of reported methods for the detection of uric acid and glucose in urine

Analyte	Sensing probe	Method	Linear range	LoD	Ref.
	nanoporous gold	electrochemical	10 – 750 µM	0.06 µM	1
	Cobalt tetroxide	colorimetric	1-10 µM, 10-600 µM	0.33 µM	9
	snowflake-like Ce-BTC@MoS$_2$	electrochemical	0.5-4.4 mM	5 µM	10
Uric acid	Carbon nanotubes with GdS-Gd2O3 nanoplates	electrochemical	0.5-30 µM, 30-2000 µM	0.380 µM	11
	graphene oxide (reduced)	electrochemical	0.200 22.0 µM	0.037 µM	12
	dextran-gold nanoparticles dual functional tablet (direct)	colorimetric	1.87 -7800 µM	3.7 µM	This work
	glucose oxidase + horseradish peroxidase dextran tablet	colorimetric	0-1 mM	0.013 mM	13
	polylactic acid and polyethylene glycol mat	electrochemical	3.4 – 5.5 mM	0.197 mM	14
Glucose	molecularly imprinted polymers	electrochemical	1.37–330 µM, 14.38–330 µM	1.37 uM, 14.38 µM	15
	hydrogel microspheres	SERS	0-25 mM	10 µM	16
	polymer nanogels – TiO$_2$ nanoparticles	colorimetric	1-7 mM	0.96 mM	17
	dextran-gold nanoparticles dual functional tablet (direct)	colorimetric	0.625 – 10 mM	0.625 mM	This work

References

1 K. Shang, S. Wang, S. Chen and X. Wang, *Biosensors (Basel)*, 2022, **12**, 588.

2 A. Jaiswal, S. Madaan, N. Acharya, S. Kumar, D. Talwar and D. Dewani, *Cureus*, 2021, **11**, 19649.

3 D. Jiang, Y. Zhu, Z. Sun, Z. Zhu, Q. He, X. Huang, Y. Yang, Y. Ge, Q. Zhang and Y. Wang, *Chemical Engineering Journal*, 2024, **486**, 150220.

4 Y. Liu and J. Liu, *Analysis & Sensing*, 2022, **2**, e202200010.

5 L. Su, J. Feng, X. Zhou, C. Ren, H. Li and X. Chen, *Anal Chem*, 2012, **84**, 5753–5758.

6 Z. Wang, S. Dong, M. Gui, M. Asif, W. Wang, F. Wang and H. Liu, *Anal Biochem*, 2018, **543**, 82–89.

7 X. Mao and C. Zhang, *Talanta*, 2022, **238**, 123052.

8 H. Yao, A. J. Shum, M. Cowan, I. Lähdesmäki and B. A. Parviz, *Biosens Bioelectron*, 2011, **26**, 3290–3296.

9 J. Cao, C. Xie, Y. Zeng and Y. Wu, *Microchemical Journal*, 2024, **204**, 111079.

10 X. Zha, Z. Zhao, C. Ding, Z. Fan and Y. Yang, *Sens Actuators B Chem*, 2025, **422**, 136474.

11 S. Verma, A. Sen, N. Dutta, P. Sengupta, P. Chakraborty and G. Dutta, *Langmuir*, 2024, **40**, 21427–21441.

12 N. Baig, A.-N. Kawde and A. Elgamouz, *Biosens Bioelectron X*, 2022, **11**, 100205.

13 H. Hajimiri, S. H. Safiabadi Tali, M. Al-Kassawneh, Z. Sadiq and S. Jahanshahi-Anbuhi, *Biosensors (Basel)*, 2023, **13**, 893.

14 N. O. Gomes, R. T. Paschoalin, S. Bilatto, A. R. Sorigotti, C. S. Farinas, L. H. C. Mattoso, S. A. S. Machado, O. N. Oliveira and P. A. Raymundo-Pereira, *ACS Sustain Chem Eng*, 2023, **11**, 2209–2218.

15 M. Caldara, J. W. Lowdon, G. van Wissen, A. G. Ferrari, R. D. Crapnell, T. J. Cleij, H. Diliën, C. E. Banks, K. Eersels and B. van Grinsven, *Adv Mater Interfaces*, 2023, **10**, 2300182.

16 Q. Wang, D. Sun, X. Ma, R. Huang, J. Xu, X. Xu, L. Cai and L. Xu, *Talanta*, 2023, **260**, 124657.

17 T. Shi, D. Kou, L. Gao, Y. Xue, S. Zhang and W. Ma, *ACS Appl Nano Mater*, 2024, **7**, 3116–3128.
